# In vitro skin culture media influence the viability and inflammatory response of primary macrophages

**DOI:** 10.1038/s41598-021-86486-7

**Published:** 2021-03-29

**Authors:** Chiara Griffoni, Berna Neidhart, Ke Yang, Florian Groeber-Becker, Katharina Maniura-Weber, Thomas Dandekar, Heike Walles, Markus Rottmar

**Affiliations:** 1 Laboratory for Biointerfaces, Empa - Swiss Federal Laboratories for Materials Science and Technology, St. Gallen, Switzerland; 2 Department Tissue Engineering & Regenerative Medicine, University Hospital Würzburg, Würzburg, Germany; 3 Translational Center for Regenerative Therapies, Fraunhofer-Institute for Silicate Research ISC, Würzburg, Germany; 4 Department of Bioinformatics, University of Würzburg, Würzburg, Germany; 5 Core Facility Tissue Engineering, Otto-Von-Guericke-University, Magdeburg, Germany

**Keywords:** Skin models, Immunological models

## Abstract

The replacement of animal models for investigation of inflammation and wound healing has been advancing by means of in vitro skin equivalents with increasing levels of complexity. However, the current in vitro skin models still have a limited pre-clinical relevance due to their lack of immune cells. So far, few steps have been made towards the incorporation of immune cells into in vitro skin and the requirements for immunocompetent co-cultures remain unexplored. To establish suitable conditions for incorporating macrophages into skin models, we evaluated the effects of different media on primary keratinocytes, fibroblasts and macrophages. Skin maturation was affected by culture in macrophage medium, while macrophages showed reduced viability, altered cell morphology and decreased response to pro- and anti-inflammatory stimuli in skin differentiation media, both in 2D and 3D. The results indicate that immunocompetent skin models have specific, complex requirements for supporting an accurate detection of immune responses, which point at the identification of a suitable culture medium as a crucial pre-requisite for the development of physiologically relevant models.

## Introduction

Application of skin models for risk and efficacy testing has gained interest and relevance since the first skin equivalent was described in 1981^1^. Since then, in vitro skin culture has greatly evolved and is now a well-established technology. Although many models have been designed to assess tissue responses to the application of irritants or upon wounding, the absence of immune cells limits their physiological relevance, underlining the need for more advanced models better mimicking human physiological responses that would lead to the replacement of animal models. In vivo, skin response to inflammation not only involves tissue-resident cells, but a range of immune cells that are recruited after pro-inflammatory signals are released at the site of injury^2^. Depending on the application, different cell types have been included in skin cultures up to the present, however, the inclusion strategies, scaffolds, cell sources, culture media and culture times are highly heterogeneous^3,4^.

Macrophages are crucial players during wound healing, being capable of both eliciting and inhibiting the progression of inflammation due to their intrinsic plasticity^5^. Because of their crucial role in tuning inflammatory responses, implementation of primary macrophages into skin equivalents is expected to greatly enhance the relevance of in vitro models by better recapitulating the physiological behavior of inflammatory and healing processes. By sensing the environment, macrophages can acquire a spectrum of distinct phenotypes through a process called polarization, whose representative extremes are pro-inflammatory or M1-like, and anti-inflammatory or M2-like phenotypes^6,7^. M1-like macrophages derive from stimulation with lipopolysaccharide (LPS) and interferon-γ (IFN-γ) and secrete pro-inflammatory cytokines and chemokines such as IL-1β, IL-6, IL-12 and tumor necrosis factor α, while M2-like macrophages are a result of IL-4 and/or IL-13 stimulation and secrete anti-inflammatory cytokines such as IL-10 and transforming growth factor β^6,7^.

To date, only very few studies have described the incorporation of macrophages into in vitro skin cultures^8,9^, reporting the detection of immune cells through immunohistological evaluation and by quantification of the secreted cytokines. However, the lack of epidermis^9^ or the absence of a healthy epidermis^8^ limits their relevance. Skin response to injury is the result of synergistic signaling between epidermis and dermis^10–12^, thus the lack of a healthy epidermis affects the signals triggered by skin tissue and the inflammatory process. Additionally, prior to the incorporation of immune cells, no evaluation of the culture conditions had been performed. The effects of culture medium on skin equivalents’ maturation have been previously reported, demonstrating that different nutrient combinations affect tissue thickness, stability of dermo-epidermal junctions and number of epidermal layers^13^. While the ideal conditions to obtain a mature epithelium differ depending on the system used for culture^14^, diverse supplements have been shown to enhance differentiation. For instance, calcium concentrations above 1 mM promote the formation of tight junctions between keratinocytes and trigger differentiation^15,16^. Serum supplementation, conversely, contains factors that both promote and inhibit keratinocyte differentiation^17,18^, which led to the use of low serum-containing^11,19–21^ or even serum-free media^22,23^ for in vitro skin culture. The generation of in vitro skin models requires a complex formulation of nutrients and growth factors, but thus far described immunocompetent skin equivalents used media suitable for skin maturation^8,9^ and neglected the potential effect of the medium on the employed immune cells. While the implementation of immune cells into skin equivalents represents a step towards the generation of more relevant in vitro models, the pre-requisites for the triple co-culture described by immunocompetent skin models have yet to be established.

We here evaluate the influence of culture medium on in vitro skin maturation as well as macrophages phenotype and functionality, to better understand the role of culture conditions on the future generation of a functional immunocompetent in vitro skin model. For this, two skin differentiation media were selected, a serum-free commercially available medium specifically designed for 3D air-lift culture of skin equivalents and a fully defined medium described in literature^19^, which demonstrated excellent skin tissue maturation and the ability to support a co-culture of skin equivalents and Langerhans cells. The effects of the selected media were compared to a commonly used medium for standard in vitro macrophage culture^24–29^. Fibroblasts and keratinocytes were evaluated for their ability to generate mature skin in the selected media, and their viability and proliferation in single 2D cultures were analyzed. Macrophages were examined for cell viability, morphology and their response to pro- and anti-inflammatory stimuli in 2D cultures. In addition, macrophages were embedded in collagen hydrogels to study the effects of the media in an in vivo-like three-dimensional environment. Cell viability, ability to respond to pro-inflammatory stimuli and ability to migrate through the collagen gels were assessed. The presented results show that skin differentiation media have inhibitory effects on the inflammatory response, and at the same time reveal that in vitro skin models have a protective effect against inflammation.

## Results and discussion

### Skin differentiation media promote skin maturation but decrease macrophage viability

**Figure 1:**
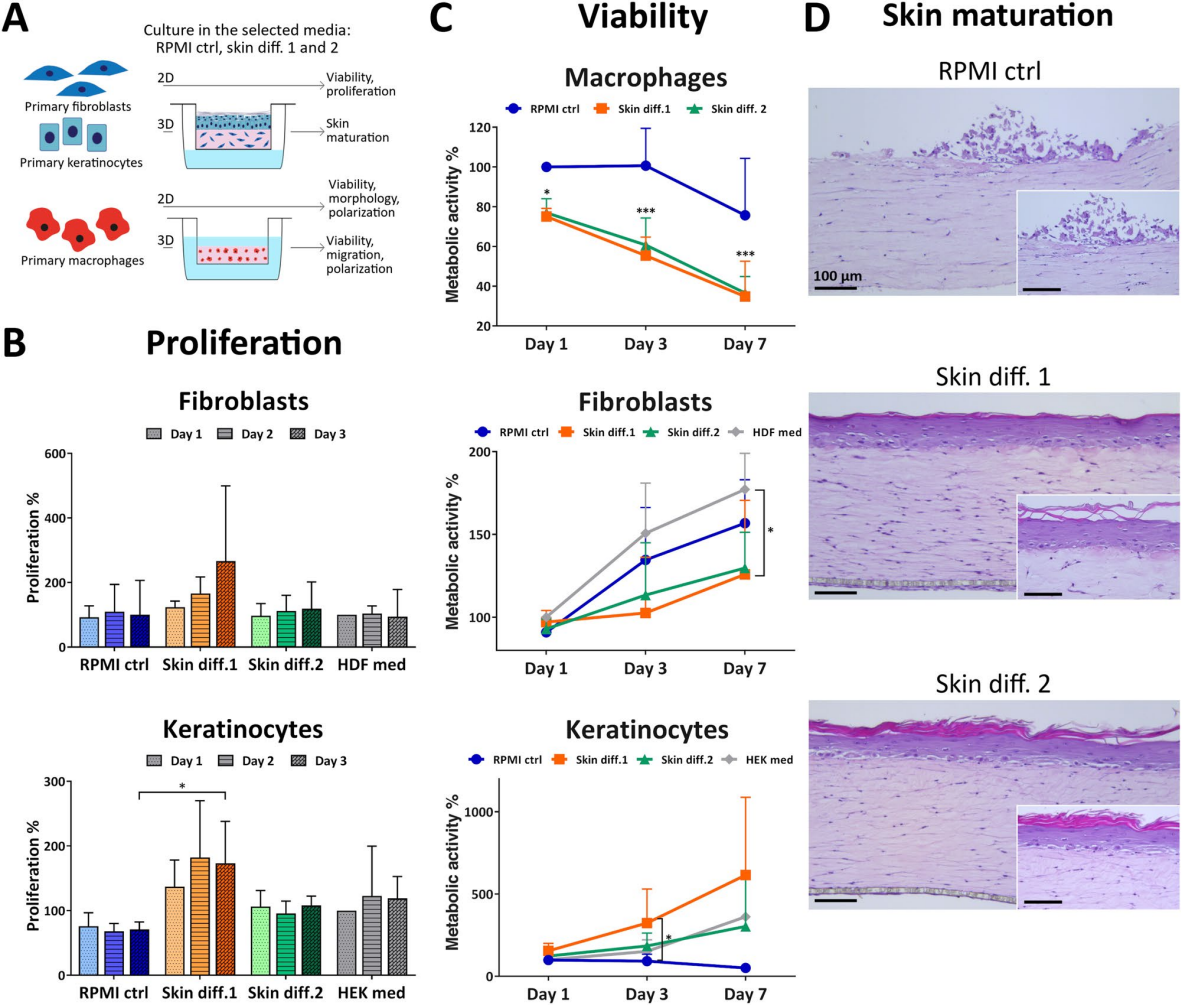
Influence of culture medium composition on skin cell viability and proliferation and in vitro skin maturation. (**A**) Schematic of the experiments performed. (**B**) Proliferation and (**C**) viability of primary fibroblasts, keratinocytes and macrophages cultured in RPMI control (“RPMI ctrl”), skin differentiation 1 (“Skin diff. 1”) or skin differentiation 2 (“Skin diff. 2”) media. Values are normalized to fibroblasts, keratinocytes or macrophages cultured for 1 day in fibroblast (“HDF med”), keratinocyte (“HEK med”) or RPMI control medium, respectively. N = 3, triplicate measurements for fibroblasts and keratinocytes; N = 5, triplicate measurements for macrophages. Error bars represent standard deviation (two-way ANOVA, Tukey's multiple comparisons test). Statistical significance indicated with *p < 0.05, ***p < 0.001. (**D**) Representative histological images of skin models cultured in the three media after 7 days of air-lift culture. N = 3, triplicate measurements. Scale bars: 100 µm.

To understand the influence of culture medium on the viability of single cell types, fibroblasts, keratinocytes and macrophages were cultured in RPMI control, skin differentiation media 1 and 2 for up to 7 days and were assessed for their metabolic activity as a measure of the number of viable cells. Additionally, proliferation of fibroblasts and keratinocytes was assessed after 1, 2 and 3 days of culture (Fig. 1A).

Fibroblast proliferation rates were increased in presence of skin differentiation medium 1, but without any statistical significance when compared to cells grown in fibroblast medium (Fig. 1B). Similarly, metabolic activity showed increased values over time for all conditions, with a significant difference (*p* < 0.05) for skin differentiation medium 1 at 7 days compared to cells grown in fibroblast medium (Fig. 1C). Proliferation of keratinocytes showed increased values for skin differentiation medium 1, while cells in RPMI control medium had the lowest values detected (Fig. 1B). Similarly, assessing metabolic activity showed increasing values over time in skin differentiation media, with a significant (*p* < 0.05) difference for skin differentiation medium 1 at 3 days compared to RPMI control medium (Fig. 1C). Notably, keratinocytes cultured in RPMI control medium displayed decreasing values over time, indicating that the medium does not support keratinocyte survival. Macrophage metabolic activity values showed a significant (*p* < 0.05 at day 1, *p* < 0.001 at days 3 and 7) decrease already after one day of culture in either skin differentiation media, with similar values for both (Fig. 1C). In contrast, culture in RPMI control medium resulted in decreased metabolic activity values only after one week of culture. The decreased macrophage viability in skin differentiation media, as indicated by the low metabolic activity values, is likely due to the absence or lower concentration of serum in skin differentiation media, as macrophages require serum for survival^30^. No other supplement present in skin differentiation medium 2 has been associated with altered macrophage viability. To enhance epithelial stratification, for instance, medium is supplemented with vitamin C^22,31–34^ or amino acids such as L-carnitine and L-serine^11,19,32,34^. The mentioned supplements are involved in the differentiation process of keratinocytes, but their effects on primary immune cells remain largely unexplored. Similarly, the only known component of skin differentiation 1, hydrocortisone, has never been associated with decreased macrophage viability. While the influence of serum or other components on macrophage survival is unknown, the significant decrease in metabolic activity values suggests that skin differentiation media do not support macrophage culture for extended periods of time.

To investigate the influence of medium on tissue maturation, skin models were cultured in RPMI control, skin differentiation medium 1 or 2 and evaluated for epidermal stratification. Culture in RPMI control medium did not promote the formation of a mature epidermis, as no stratified keratinocyte layers were detected (Fig. 1D). Only few keratinocytes were visible on top of the dermal compartments, likely due to lack of cell attachment, spreading and proliferation because of insufficient survival cues provided by the medium. Despite the high serum content of RPMI control medium, no other supplement such as KGF or CaCl_2_ is present to promote the adhesion of cells to the collagen gel and to sustain keratinocyte proliferation and differentiation (Tab. S1). Those results reflect the effects observed for 2D culture in RPMI control medium, which showed a decrease in cell viability of 50% after 7 days and low proliferation rates, confirming that also in 3D this medium does not support keratinocyte culture. In contrast, the use of skin differentiation media resulted in epidermal stratification, as indicated by the presence of several layers of keratinocytes and of a stratum corneum on the apical part of the epidermis (Fig. 1D). Skin differentiation medium 1 is a serum-free medium specifically designed for the co-culture of fibroblasts and keratinocytes, and skin differentiation medium 2 contains KGF, CaCl_2_ and other supplements. Conversely, RPMI control medium does not contain any specific factor promoting the survival and proliferation of keratinocytes, at the same time including a high serum percentage that interferes with differentiation. These findings are in agreement with previous results from Black et al.^33^, who demonstrated that the presence of serum during air-lift culture prevents the maturation of in vitro skin models.

### Skin differentiation media affect macrophage morphology and response to stimuli

To understand whether skin media are suitable for macrophage culture, morphology was examined for 7 days and the cells‘ ability to polarize towards M1-like and M2-like phenotypes after cytokine stimulation was investigated.

**Figure 2:**
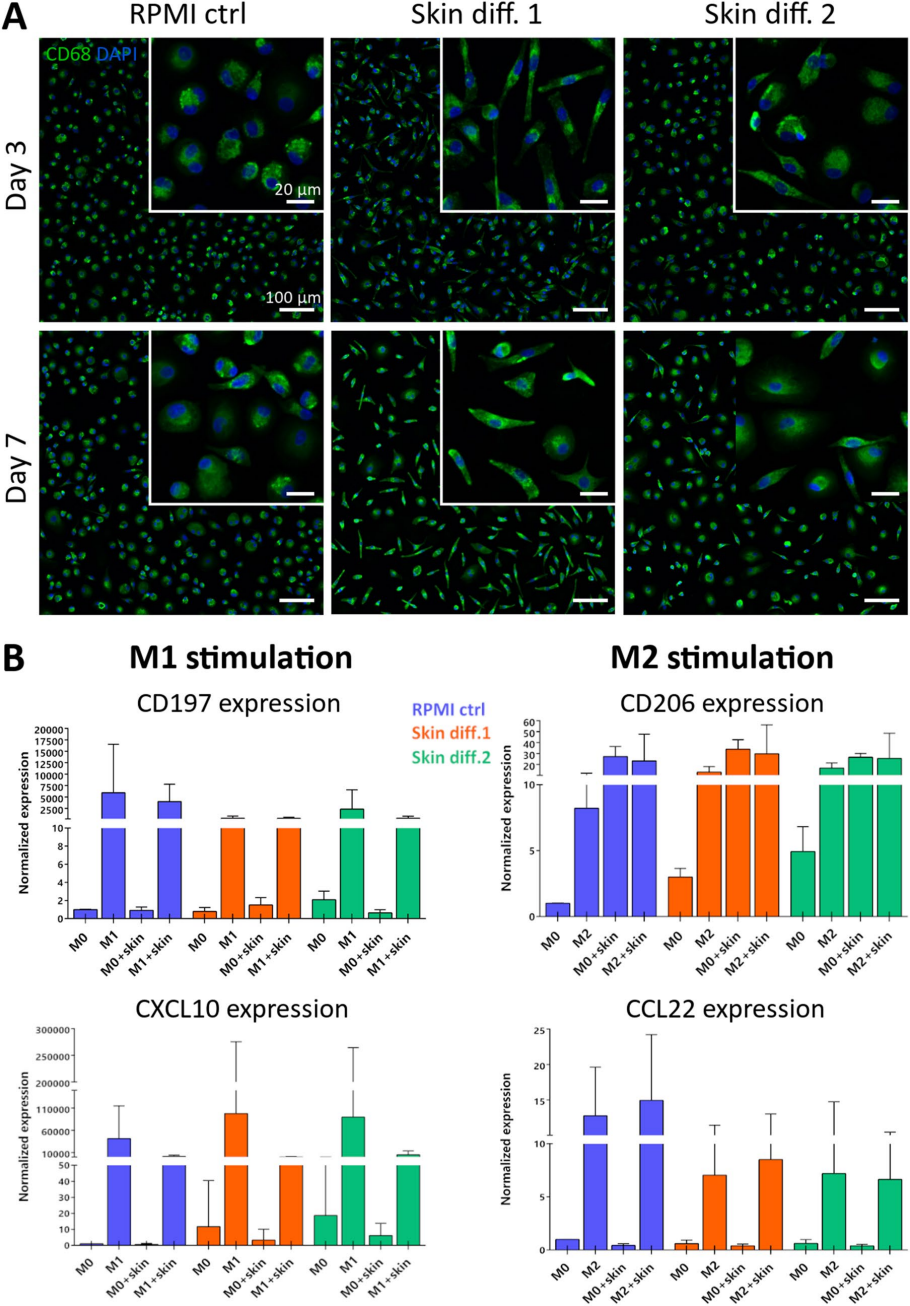
Influence of culture medium on primary macrophage morphology and gene expression. (**A**) Representative images of macrophages cultured for 3 and 7 days in RPMI control (“RPMI ctrl”), skin differentiation 1 (“Skin diff. 1”) or skin differentiation 2 (“Skin diff. 2”) media upon staining with CD68 and DAPI. N = 3, duplicate measurements. Scale bars: 100 µm for the lower magnification, 20 µm for the higher magnification (insets). (**B**) Quantification of gene expression of primary macrophages either unstimulated (“M0”) or polarized into M1- or M2-like phenotypes in the three media, in presence ("M1 + skin"; "M2 + skin") or absence ("M1"; "M2") of skin as a co-culture. M1-like stimulated cells were analyzed for M1 markers CD197 and CXCL10, and M2-like stimulated cells for M2 markers CD206 and CCL22. N = 10, duplicate measurements for macrophages cultured alone; N = 5, single measurement for macrophage-skin co-cultures. Error bars represent standard deviation.

Already after 3 days, cell morphology was affected by culture in skin differentiation media. In RPMI control medium, cells showed a round-shaped morphology, while in skin media they appeared elongated and spindle-shaped, which was more evident in presence of skin differentiation medium 1 (Fig. 2A). After 7 days of culture, the same tendency as well as qualitatively a brighter staining intensity of CD68 was observed in presence of skin medium 1. An elongated cell shape and a brighter CD68 staining indicate opposite effects of the skin media on cell polarization. In fact, an upregulated CD68 expression was reported during inflammation^35,36^, indicating macrophage polarization to a pro-inflammatory or M1 phenotype. At the same time, macrophages with an elongated morphology were correlated with an anti-inflammatory or M2 phenotype^37^.

The results observed in macrophage morphology evaluation were further investigated by means of gene expression analysis. Specific M1 and M2 markers were quantified after culture in the different media, both in unstimulated cells (Fig. S1) and in response to M1 and M2 stimulation (Fig. S2). Macrophage gene expression was measured both for monocultures and for macrophages in presence of 3D skin equivalents as a co-culture (Fig. 2B). Both skin differentiation media showed to affect the expression of pro-inflammatory markers in macrophages monocultures, both in unstimulated cells and upon stimulation with LPS and IFN-γ. Values of the M1 marker CD197 were decreased in skin differentiation media 1 and 2, whereas CXCL10 showed an increased expression. Gene expression of M2 markers CD206 and CCL22 showed the same trend in both skin differentiation media, both for unstimulated macrophage monocultures and upon anti-inflammatory stimulation with IL-4, with an increased CD206 expression and a decreased expression of CCL22 (Fig. 2B).

To exclude that the observed differences in gene expression resulted from the M-CSF supplementation during the monocyte-to-macrophage differentiation, gene expression of cells generated with granulocyte–macrophage colony-stimulating factor (GM-CSF) supplementation was also evaluated. Indeed, M-CSF has been shown to steer cell fate towards M2-like cells, while GM-CSF was indicated to steer towards a M1-like phenotype^27^. The results did not indicate that M1 and M2 expression was dependent on the monocyte-to-macrophage differentiation conditions (Fig. S3), pointing at the composition of skin differentiation media to be the cause of such effects. Isoproterenol and hydrocortisone, both contained in skin differentiation medium 2, are known to downregulate the inflammatory response to LPS in macrophages^38,39^. Skin differentiation medium 1 to our knowledge contains hydrocortisone, which could explain the decreased inflammatory response. Other supplements present in skin differentiation medium 2 have not been linked to altered immune cellular responses.

In vivo, monocytes and macrophages are under a continuous stimulation from cytokines, chemokines and growth factors constitutively secreted by skin tissue to maintain homeostasis^2,40^. Mimicking this situation, macrophages were co-cultured with 3D skin equivalents to investigate the influence of skin paracrine signaling on the response of macrophages to stimuli. Macrophage morphology did indicate an altered response to stimuli, with less rounded and spread cells in presence of 3D skin equivalents (Fig. S4). Gene expression quantification confirmed that the presence of skin as a co-culture with macrophages influenced the expression of the analyzed genes (Fig. 2B). M1 markers CD197 and CXCL10 were down-regulated, indicating a further inhibition of macrophages ability to respond to pro-inflammatory stimuli in presence of skin equivalents. At the same time, the M2 marker CD206 showed an upregulation when skin was present as a co-culture, suggesting an enhanced response to anti-inflammatory stimuli (Fig. 2B). The changes in gene expression levels upon polarization with pro- and anti-inflammatory stimuli were statistically not significant for macrophages cultured alone and in co-culture with 3D skin equivalents (Fig. S2), however showed statistically significant differences in unstimulated cells (Fig. S1). Due to the different gene expression upregulation or downregulation patterns within the M1 markers CD197 and CXCL10 and within the M2 markers CD206 and CCL22, we could not confirm the correlation between cell shape and polarization state. Compared to other studies where the connection between cell morphology and function was investigated^37,41^, the present study employed human primary blood monocytes-derived macrophages. The use of primary immune cells implies a relevant donor-to-donor variation, dependent on factors as the health state at the moment of isolation, which is reflected on the detected heterogeneous cellular response to inflammation^28^. The results obtained from macrophage-skin co-cultures are in agreement with recent findings by Limandjaja et al.^42^, who demonstrated that the co-culture of monocytes below in vitro skin equivalents results in the differentiation of the immune cells towards an M2 phenotype. While the methods to investigate macrophage phenotype differ from the present study, the medium composition used in Limandjaja et al.^42^ for the co-culture is comparable to skin differentiation medium 2, as it was a 3:1 ratio combination of DMEM/Ham’s F12 initially supplemented with serum, KGF, insulin, hydrocortisone and isoproterenol, and after 3 days further supplemented with L-carnitine, L-serine, ascorbic acid, and other components. The observed anti-inflammatory effect exhibited from skin equivalents further strengthens the correlation between in vitro skin and human skin, where resident skin cells participate in immunosurveillance to protect the body from infection^43^.

### Skin differentiation media influence viability, response to stimuli and migration of 3D-embedded macrophages

In an immunocompetent skin model, macrophages would be cultured within a 3D environment. Therefore, cells were embedded in collagen gels to study the influence of a 3D environment on viability, response to pro-inflammatory stimuli and ability to migrate in different media compositions.

**Figure 3:**
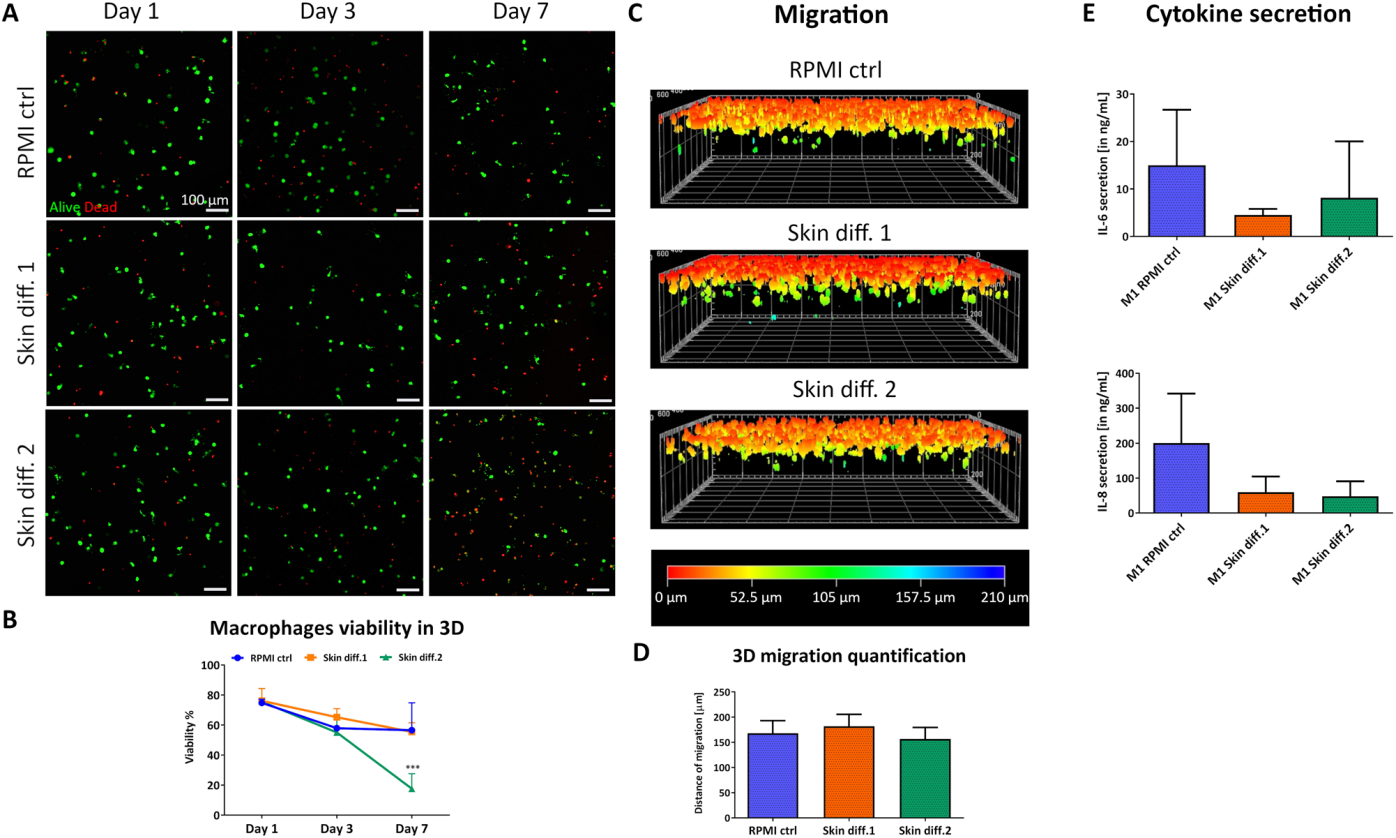
Influence of culture medium composition on viability, response to pro-inflammatory stimuli and migration ability of macrophages in a 3D environment. (**A**) Representative maximum intensity projections of macrophages after culture in RPMI control (“RPMI ctrl”), skin differentiation 1 (“Skin diff. 1”) or skin differentiation 2 (“Skin diff. 2”) media, upon live/dead staining. Scale bars: 100 µm. (**B**) Quantification of macrophage viability. N = 3, n = 2. Error bars represent standard deviation. Statistical significance indicated with ***p < 0.001. (**C**) Representative 3D reconstructed images of live/dead staining after vertical invasion assay, color coded for invasion depth. (**D**) Quantification of macrophage maximal migration distance. N = 3, triplicate measurements. Error bars represent standard deviation. (**E**) Quantification of pro-inflammatory cytokines IL-6 and IL-8 secretion in supernatants. N = 3, duplicate measurements. Error bars represent standard deviation.

Macrophage viability showed a small decrease over 7 days, with a similar trend for all media until day 3 (Fig. 3A,B). However, by day 7, a large number of dead cells could be observed in skin differentiation medium 2, with viability decreasing below 20%. Surprisingly, skin differentiation medium 2 showed to negatively influence macrophages survival to a greater extent in 3D compared to 2D cultures. The effects are plausible due to the presence of a 3D matrix around the cells that prevented dead cells to be removed from the area through media change, increasing the dead cell fraction compared to the 2D setting.

The ability of macrophages to migrate through the tissue is a crucial requirement during the inflammatory response, therefore we performed a vertical invasion assay. Macrophages showed the ability to migrate through the dense collagen matrix in all conditions (Fig. 3C), with a maximal migration depth of 182 ± 28 µm, 157 ± 22 µm and 168 ± 21 µm for skin differentiation medium 1, medium 2 and control, respectively (Fig. 3D). While no statistically significant differences in migration depth could be observed, a different number of cells attached to the surface of the gel prior migration. In fact, observation of cell distribution on the collagen gel surface showed that a lower number of cells adhered in presence of skin differentiation medium 2, compared to gels cultured in RPMI control and skin differentiation medium 1 (Fig. S5). The encapsulation of macrophages in collagen gels did not affect their ability to respond to pro-inflammatory stimuli, as IL-6 and IL-8 could be detected in all supernatants upon stimulation. However, lower amounts of both cytokines were detected in the supernatants of gels cultured in the two skin differentiation media (Fig. 3E). Despite the lack of statistical significance, the detected values followed the same trend as the gene expression levels of 2D-cultured cells with the respective polarization stimuli (Fig. 2B), pointing towards an inhibition of the pro-inflammatory response in presence of both skin differentiation media.

### Macrophage medium has a detrimental effect on mature skin equivalents

As the use of skin differentiation media affects macrophage viability and response to inflammatory stimuli, the feasibility of culturing immunocompetent skin in RPMI control medium was evaluated for the theoretical duration of a potential inflammatory response. For this, fully differentiated skin equivalents grown in skin differentiation medium 1 or 2 were transferred into RPMI control medium and cultured for further 7 days, and potential changes to the skin structure were evaluated by immunohistochemistry.

**Figure 4:**
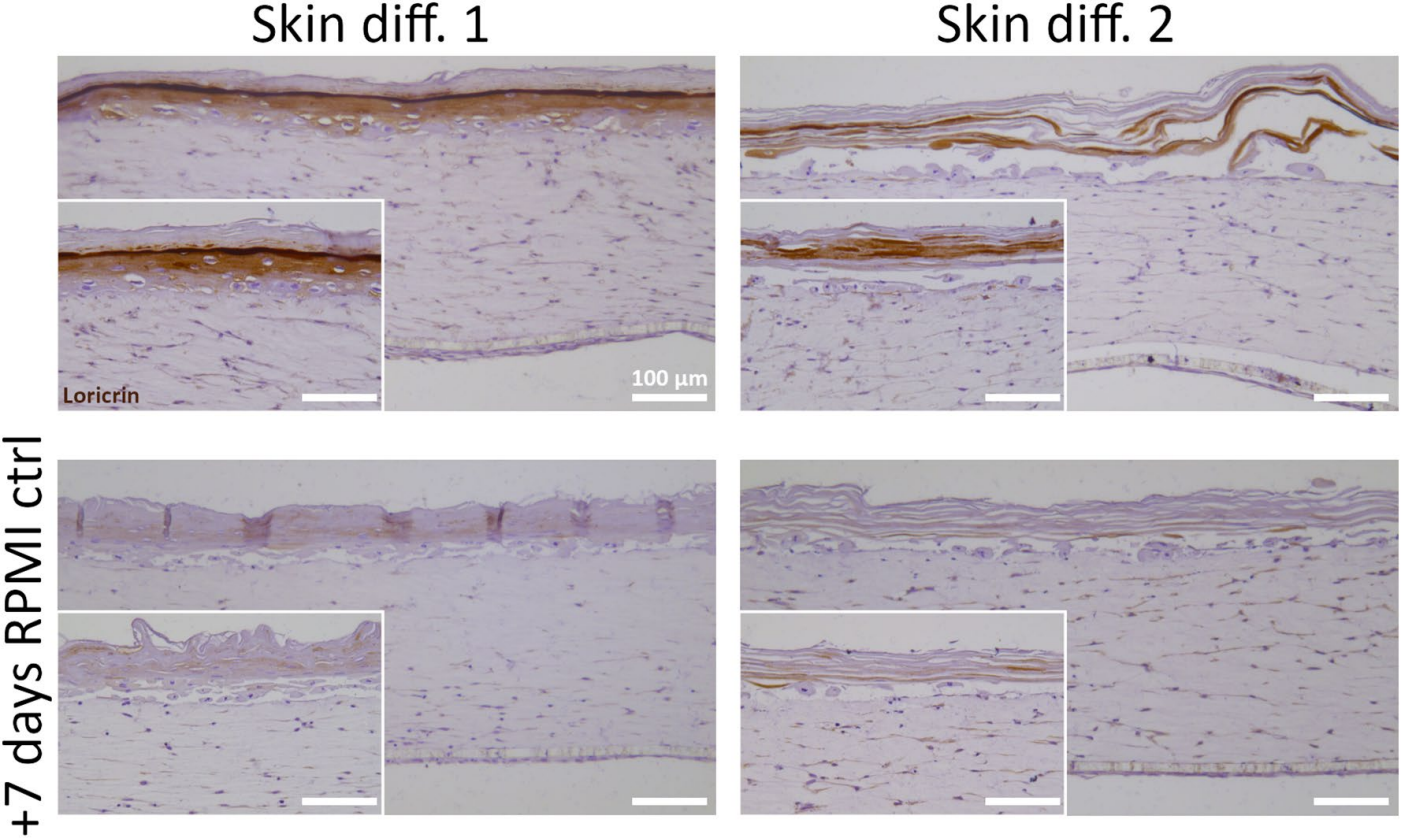
Evaluation of macrophage medium effects on epidermal structure of skin models. In vitro skin equivalents were generated by culture in skin differentiation medium 1 (“Skin diff. 1”) or skin differentiation medium 2 (“Skin diff. 2”) for 14 days at air-lift culture (upper row), and then further cultured for 7 days in RPMI control medium (“RPMI ctrl”) (lower row). Immunohistochemistry against loricrin was performed and counterstaining with hematoxylin followed. N = 2, triplicate measurements. Scale bars: 100 µm.

Culture in RPMI control medium was already shown to prevent in vitro skin maturation (Fig. 1D), but also shifting to RPMI control medium after full skin epidermalization had a detrimental effect on skin structure. The expression of the late differentiation marker loricrin was detected in skin equivalents differentiated for 14 days in skin differentiation medium 1 or 2. However the shift to RPMI control medium showed no expression of the differentiation marker (Fig. 4). The negative effects on skin structure observed after RPMI control medium culture are likely due to the drastic reduction of supplements promoting cell survival and differentiation, as skin differentiation media contain elements promoting 3D skin culture while RPMI control medium is only supplemented with serum. As skin differentiation medium 1 has a proprietary composition, only the nutrients present in skin differentiation medium 2 and RPMI control medium could be compared (Tab. S1). Calcium concentration is higher in skin differentiation medium 2 compared to RPMI control medium, and since it has been linked with the promotion of epidermal stratification^15–17^, its deprivation could be involved in degeneration of the epidermal structure. The higher serum content of RPMI control medium may also influence epidermal structure, as serum contains factors that inhibit keratinocyte differentiation^18,44^. The percentage of serum in RPMI control medium is 10%, compared to 1.25% in skin differentiation medium 2 and serum-free conditions in skin differentiation medium 1. Differently than RPMI control, whose basal medium is supplemented only with serum and antibiotics, skin differentiation medium 2 is supplemented with different compounds to support tissue viability in culture. Among these, KGF has a crucial role in maintaining keratinocytes proliferation and thus differentiation^45^, therefore its deprivation could have negatively affected basal keratinocytes turnover. Overall, a combination of all those factors is the probable cause of the negative effects observed on differentiation after medium change to RPMI control.

The generation of advanced immunocompetent skin models better mimicking the human physiology is a pivotal step in the replacement of animal use in research. The central role of macrophages during inflammation has advanced their inclusion into in vitro skin equivalents, however few models have been described^8,9^ and several aspects for the generation of functional immunocompetent skin remain unresolved^3^. The optimization of a co-culture medium is a crucial step towards the development of any functional model, as each cell type requires specific nutrients to remain functional. The investigation of two culture media designed for in vitro skin maturation on primary macrophages showed that their inflammatory response is affected by this combination of nutrients. Macrophages showed reduced viability, altered cell morphology and altered response to pro- and anti-inflammatory stimuli both in a 2D and 3D environment, indicating that the identification of a culture medium supporting both skin maturation and macrophage functionality is a crucial step towards the generation of immunocompetent skin.

Further investigation is needed to determine which components affect immune cells and skin maturation, to select the key elements needed for maintenance of immunocompetent skin models. For this however, not only gene expression data, but also protein expression should be assessed, as the abundance of proteins can be affected by translational and post-translational regulation. In parallel to the identification of these crucial components, the substitution of animal-derived products currently in use for the fabrication of in vitro skin equivalents represents another step towards the total replacement of animals in research. While the current standard in vitro 3D cell culture setup is mainly represented by the use of animal-derived products such as serum and collagen, research on alternatives that do not require animal involvement is ongoing.

## Methods

### Cell isolation and culture

Primary human dermal fibroblasts pooled from several donors and juvenile keratinocytes isolated from a single donor were purchased from CELLnTEC (CELLnTEC Advanced Cell Systems AG, Bern, Switzerland). Fibroblasts were cultured in DMEM supplemented with 10% fetal bovine serum (FBS), 1% L-glutamine and 1% penicillin–streptomycin-neomycin (PSN), also referred to as “HDF medium”. As recommended by the supplier, fibroblasts were used until passage 8 with a seeding density of 1000 cells/cm^2^, passaging the cells every 6–7 days. Keratinocytes were maintained in CnT-prime epithelial culture medium (CELLnTEC Advanced Cell Systems AG), referred to as “HEK medium” and used until passage 6. Passaging was performed every 5 days, seeding the cells at a density of 4000 cells/cm^2^ as recommended by the supplier. Primary monocytes were isolated from peripheral blood of healthy blood donors who provided informed consent. For each experiment, a different donor was recruited. Approval from the local committee of swissethics (Swiss Association of Research Ethics Committees, BASEC Nr PB_2016_00816) in accordance to relevant guidelines and regulations was obtained. Freshly drawn heparinized blood was first was first diluted 1:1 in a solution containing 2% FBS + 1 mM EDTA in PBS and then separated into the different blood components by Lymphoprep (STEMCELL Technologies, Cologne, Germany) density gradient centrifugation. The white blood cell fraction, visible as a white ring between the plasma components and the Lymphoprep, was carefully collected and washed twice with PBS to avoid carryover of solvents into the following steps. To purify and enrich the monocytic population, specific negative selection was performed using the Monocyte Isolation Kit II (Miltenyi Biotec, Bergisch Gladbach, Germany) according to the manufacturer’s instructions. Purified monocytes were seeded at a density of 62,000 cells/cm^2^ in monocyte differentiation medium, consisting of Roswell Park Memorial Institute medium (RPMI 1640) (Sigma-Aldrich) supplemented with 10% FCS, 1% PSN and 20 ng/mL of macrophage colony-stimulating factor (M-CSF) (Thermo Fisher Scientific, Schwerte, Germany). After 6 days, differentiated macrophages were detached by incubation in TrypLE Express (Thermo Fisher Scientific) for 20 min at 37 °C, followed by gentle scraping with a cell scraper. Collected cells were resuspended in fresh macrophage medium (RPMI 1640 supplemented with 10% FCS and 1% PSN, also referred to as "RPMI control medium"), counted and used for subsequent experiments.

### Cell viability and proliferation

Cell viability was evaluated with an MTS assay (Promega, Madison, USA) and cell proliferation was analyzed using the BrdU-based Cell Proliferation ELISA kit (Roche, Basel, Switzerland), according to the manufacturer’s instructions. Three independent experiments with triplicate measurements were performed for fibroblasts and keratinocytes, 5 independent experiments with triplicate measurements for macrophages per condition.

### 2D macrophage analysis

Macrophage polarization was obtained by supplementing the medium with 100 ng/mL LPS and 20 ng/mL IFN-γ (Miltenyi Biotec) for M1-like cells and with 20 ng/mL IL-4 (Miltenyi Biotec) for M2-like cells. For this, macrophages were seeded at a concentration of 95,000 cells/cm^2^ in 24-wells plates for 24 h. CD68 (Cluster of Differentiation 68) staining was performed by incubating fixed macrophages with a 1:100 dilution of the mouse monoclonal antibody (ab955 from Abcam, Cambridge, United Kingdom) in a solution of 0.1% bovine serum albumin (BSA) in PBS at 4 °C overnight. On the following day, the secondary anti-body Alexa Fluor 488 conjugate (Invitrogen, Carlsbad, USA) was incubated for 1 h as a 1:500 dilution. 4′,6-diamidino-2-phenylindole (DAPI) was used to counterstain cell nuclei, supplemented as a 1:1000 solution in 0.1% BSA. All supplements were purchased from Sigma-Aldrich unless stated otherwise. CD68 stainings included 3 independent experiments with a minimum of 2 technical replicates each.

### Gene expression

To analyze gene expression of macrophages after polarization, RT-PCR was performed for M1- and M2-specific genes. RNA isolation was performed using the RNeasy Micro Kit (Qiagen, Hilden, Germany) according to the manufacturer’s instructions. RNA concentration and purity were then measured with a NanoDrop ND-1000 spectrophotometer (Thermo Fisher Scientific). The total RNA isolated from macrophage cultures was then reverse-transcribed using iScript cDNA synthesis Kit (Bio-Rad, Hercules, USA), using 150 ng of RNA per sample and a volume of 15 μL per reaction mix. Quantification of gene expression was performed with the iQ SYBR Green Supermix (Bio-Rad), using Glyceraldehyde 3-phosphate dehydrogenase (GAPDH) as a reference gene, and analyzing the markers CD197, CXCL10, CD206 and CCL22 (Table 1). Each primer was added at a concentration of 10 μM, adjusting the final reaction volume to 15 μL with RNase-free water. The thermal cycler was set to 95 °C for 3 min, followed by 39 cycles of 10 s at 95 °C and 30 s at 57 °C. The final steps were performed at 95 °C for 10 s, at 65 °C for 5 s, and then at 95 °C. Calculations for the quantification were performed using the 2^−ΔΔCT^ method, normalizing all values to unstimulated samples cultured in macrophage medium. Gene expression analysis was performed on 5 independent experiment, each with a minimum of 2 technical replicates.

**Table 1:** Sequence of the primers used for quantification of macrophage gene expression.

Gene	Primer name	Sequence 5′ → 3′
Glyceraldehyde-3-phosphate dehydrogenase, GAPDH	GAPDH-forward	AGTCAGCCGCATCTTCTTTT
	GAPDH-reverse	CCAATACGACCAAATCCGTTG
C–C chemokine receptor type 7, CCR7 (CD197)	CD197-forward	GTGGTTTTACCGCCCAGAGA
	CD197-reverse	CACTGTGGTGTTGTCTCCGA
C-X-C motif chemokine 10, CXCL10	CXCL10-forward	CAGTCTCAGCACCATGAATCAA
	CXCL10-reverse	CAGTTCTAGAGAGAGGTACTCCTTG
C–C motif chemokine 22, CCL22	CCL22-forward	GCGTGGTGTTGCTAACCTTC
	CCL22-reverse	CCACGGTCATCAGAGTAGGC
Mannose receptor C type 1, MRC1 (CD206)	CD206-forward	GCTACCCCTGCTCCTGGTTT
	CD206-reverse	CGCAGCGCTTGTGATCTTCA

### 3D macrophage viability and polarization

Macrophage viability and polarization in a 3D environment were evaluated after seeding 75,000 cells per collagen gel, following the same mixing procedure described above with the difference that the cell suspension was added to the collagen mixture prior to polymerization, to embed the cells within the gel. 300 µL of macrophage-containing solution were placed into each Greiner ThinCert insert for 12-well plates with transparent membrane (3 µm pore size, Greiner) and let polymerize. Polarization was induced by supplementing the culture medium with cytokines, using the same concentrations described in “2D macrophage analysis”. For viability assessment, the gels were cultured for 7 days with a medium change every 2–3 days, carefully avoiding to touch the surface of the gel. Cells were visualized after a live/dead assay (Sigma-Aldrich) staining, according the manufacturer’s instructions. Cytokine quantification was performed by collecting 1 mL of supernatant after 24 h of polarization and using human IL-6 and IL-8 ELISA kits (Thermo Fisher Scientific), following the manufacturer’s instructions. All the 3D experiments were performed on 3 independent experiments, each with a minimum of 2 technical replicates.

For the conditioned media experiments, first macrophages were seeded in 12-wells plates, following the same procedure as for the 2D macrophage seeding. Then, a ThinCert insert containing a skin equivalent was placed in each well to allow paracrine signaling between skin tissue and immune cells. After 24 h of skin-macrophages co-culture, the ThinCert inserts were removed and macrophage gene expression was quantified. Three independent experiments were performed.

Macrophage viability quantification was performed using ImageJ software, manually counting live and dead cells. A total of 5 images per condition were analyzed at each time point, with an average cell count of 90 cells per sample. Macrophage migration quantification was performed using ImageJ software, and a total of 21 images per condition were analyzed.

### 3D macrophage migration

For the vertical migration assay, ThinCert inserts for 24-well plates with transparent membrane (3 µm pore size, Greiner, Kremsmünster, Austria) were used. FibriCol bovine collagen type I (10 mg/mL, Advanced BioMatrix, San Diego, USA) was mixed with 10 × DMEM, neutralized with 0.5 M sterile filtered NaOH, and supplemented with sterile filtered water to obtain a ratio of collagen:10xDMEM:other components (water + NaOH) of 8:1:1. 150 µL per insert were added and after polymerization, macrophages were seeded on top of the gels at a concentration of 22,000 cells/cm^2^ and cultured for 48 h in the different media.

For visualization of 3D-embedded macrophages, the collagen gels containing the immune cells were stained with a live/dead assay (Sigma-Aldrich), according the manufacturer’s instructions. All images were acquired with a LSM 780 confocal laser scanning microscope (Carl Zeiss, Oberkochen, Germany), using the ZEN 2012 software (Carl Zeiss). For analysis of cell viability in 3D, each displayed image is the combination of Z-stacks obtained from a total sample thickness of 192 μm, obtained through the maximum intensity projection tool provided by the software. The vertical migration pictures were obtained by elaborating the captured images with the 3D reconstruction tool provided in the ZEN 2012 software. For all samples, ethidium homodimer-1 signal was adjusted to a negative control for each time point, incubating a sample with a solution of 0.1% digitonin.

### Generation of skin model

The dermis was generated by embedding fibroblasts in a collagen gel, as described in “3D macrophage migration, viability and polarization”, seeding 75,000 cells per sample. After polymerization, the constructs were cultured in submerged conditions in fibroblast medium for 7 days, changing the medium every 2–3 days and carefully avoiding to touch the surface of the gel. On day 7, medium was removed and the surface of each gel was coated with 50 µL of a 50 µg/mL fibronectin solution in PBS (Sigma-Aldrich) for 30 min at 37 °C. Subconfluent keratinocytes were harvested and seeded on top of the fibronectin-coated gels at a concentration of 225,000 cells/cm^2^ in keratinocyte seeding medium (DMEM/Ham’s F-12 in a 3:1 ratio, supplemented with 5% FCS, 1% PSN, 2 ng/mL keratinocyte growth factor (KGF), 1 µM hydrocortisone, 1 µM isoproterenol and 0.1 µM insulin) or in CnT-Prime Airlift medium (CELLnTEC Advanced Cell Systems AG). After 5 days of culture with a medium change at day 1 and day 3, the samples were raised to the air–liquid interface by transferring the gel-containing insert in deep-well 12-wells plates (Greiner), where 4 mL of differentiation medium were added below each insert and changed every 4 days for 2–3 weeks. The two differentiation media used were CnT-Prime Airlift medium, referred to as "Skin differentiation medium 1", or a mixture of DMEM/Ham’s F-12 in a 3:1 ratio, supplemented with 1.25% FCS, 1% PSN, 2 ng/mL KGF, 1 µM hydrocortisone, 1 µM isoproterenol, 0.1 µM insulin, 0.1 µM L-carnitine, 0.01 M L-serine and 50 µg/mL of ascorbic acid, referred to as "Skin differentiation medium 2". All supplements were purchased from Sigma-Aldrich unless stated otherwise. Each experiment was performed a minimum of 3 times, analyzing 3 technical replicates per condition.

### Histological analysis

Skin samples were fixed overnight in a solution of 4% buffered formalin, then dehydrated by incubation in increasing ethanol concentrations, washed in xylene and embedded in paraffin blocks. Afterwards, 5 µm thick sections were cut, and deparaffinization in xylene and rehydration in decreasing ethanol concentrations followed. Rehydrated samples were then stained with hematoxylin (HistoLab, Askim, Sweden) and eosin, rinsing the tissue in distilled water between the two steps. For immunohistochemistry, rehydrated slides were first incubated with a 1:500 diluted rabbit monoclonal loricrin antibody (ab176322 from Abcam) in 5% goat serum in PBS overnight at 4 °C. Then, a 1:100 solution of secondary POD-conjugated antibody was incubated for 1 h at room temperature. Loricrin staining development was performed with 3,3′- Diaminobenzidine (Dako, Santa Clara, USA) and counterstaining was performed by incubation in hematoxylin for 1 min. All supplements were purchased from Sigma-Aldrich unless stated otherwise. Each experiment was performed a minimum of 2 times, analyzing 3 technical replicates per condition.

### Statistical analysis

The GraphPad Prism software (GraphPad, San Diego, USA) was used to analyze all data. Data obtained from 3D-embedded macrophages polarization and migration were analyzed using Kruskal–Wallis test, with a Dunn post-hoc test for multiple comparisons assuming a non-parametric distribution. For all other data, two-way ANOVA with a Tukey post-hoc test for multiple comparisons was used. Statistical significance was assumed at *p* < 0.05.

## Supplementary Information

Supplementary Information
