# Supplementary material for: In vitro skin culture media influence the viability and inflammatory response of primary macrophages
Source: Sci Rep. 2021 Mar 29;11:7070. doi: 10.1038/s41598-021-86486-7 (PMC8007571; doi:10.1038/s41598-021-86486-7)
Supplement: Supplementary file 1 — Supplementary Information [file 41598_2021_86486_MOESM1_ESM.docx]

**Supplementary Information**

**In vitro skin culture media influence the viability and inflammatory response of primary macrophages**

Chiara Griffoni^1,2^, Berna Neidhart^1^, Ke Yang^1^, Florian Groeber-Becker^2,3^, Katharina Maniura-Weber^1^, Thomas Dandekar^4^, Heike Walles^3,5^, Markus Rottmar^1,^*

^1^Laboratory for Biointerfaces, Empa - Swiss Federal Laboratories for Materials Science and Technology, St. Gallen, Switzerland

^2^Department Tissue Engineering & Regenerative Medicine, University Hospital Würzburg, Würzburg, Germany

^3^Translational Center for Regenerative Therapies, Fraunhofer-Institute for Silicate Research ISC, Würzburg, Germany

^4^Department of Bioinformatics, University of Würzburg, Würzburg, Germany

^5^Core Facility Tissue Engineering, Otto-von-Guericke-University, Magdeburg, Germany

*Corresponding Author markus.rottmar@empa.ch

**
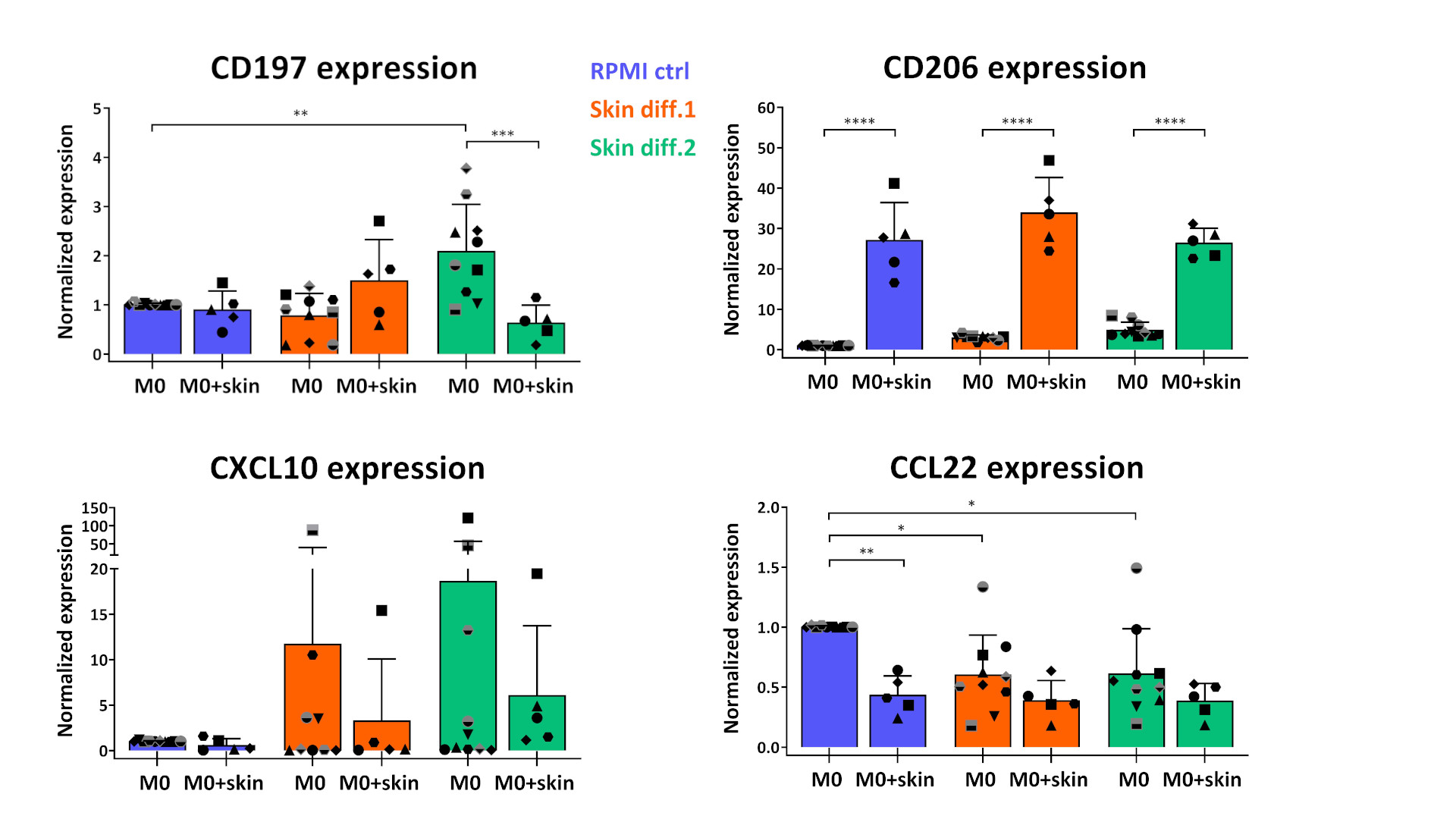
**

**Supplementary Figure S1.** Quantification of gene expression of primary macrophages in RPMI control (“RPMI ctrl”), skin differentiation 1 (“Skin diff. 1”) or skin differentiation medium 2 (“Skin diff. 2”), in presence ("M0+skin") or absence ("M0") of skin as a co-culture. The analyzed genes were M1 markers CD197 and CXCL10 and M2 markers CD206 and CCL22. All values are normalized to samples cultured in RPMI control medium, and GAPDH was used as reference gene. Each experiment represents macrophages derived from a different blood donor, each indicated with a different symbol. N=10, duplicate measurements for macrophages cultured alone; N=5, single measurements for macrophages-skin co-cultures. Error bars represent standard deviation (two-way ANOVA, Tukey's multiple comparisons test). Statistical significance is indicated with *p<0.05, **p<0.01, ***p<0.001, ****p<0.0001.


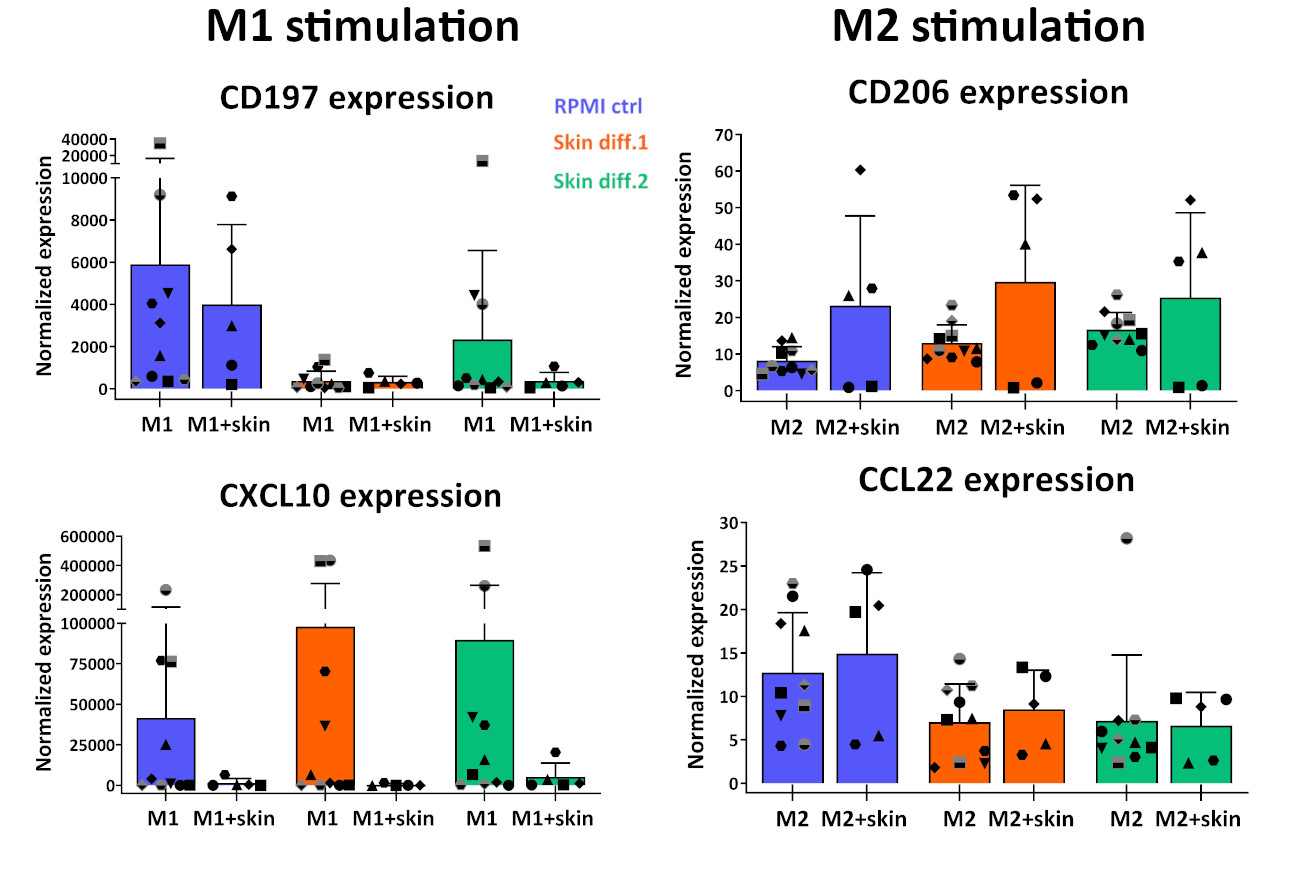


**Supplementary Figure S2**. Influence of culture medium composition on primary macrophage gene expression after polarization into M1- or M2-like phenotypes. Cells were polarized into M1- or M2-like phenotypes in RPMI control (“RPMI ctrl”), skin differentiation 1 (“Skin diff. 1”) or skin differentiation medium 2 (“Skin diff. 2”), in presence ("M1+skin"; "M2+skin") or absence ("M1"; "M2") of skin as a co-culture. M1-like stimulated cells were analyzed for M1 markers CD197 and CXCL10, and M2-like stimulated cells were analyzed for M2 markers CD206 and CCL22. All values are normalized to unstimulated samples cultured in RPMI control medium, and GAPDH was used as reference gene. Each experiment was performed with macrophages derived from a different blood donor, indicated with different symbols. N=10, duplicate measurements for macrophages cultured alone; N=5, single measurements for macrophages-skin co-cultures. Error bars represent standard deviation (two-way ANOVA, Tukey's multiple comparisons test).


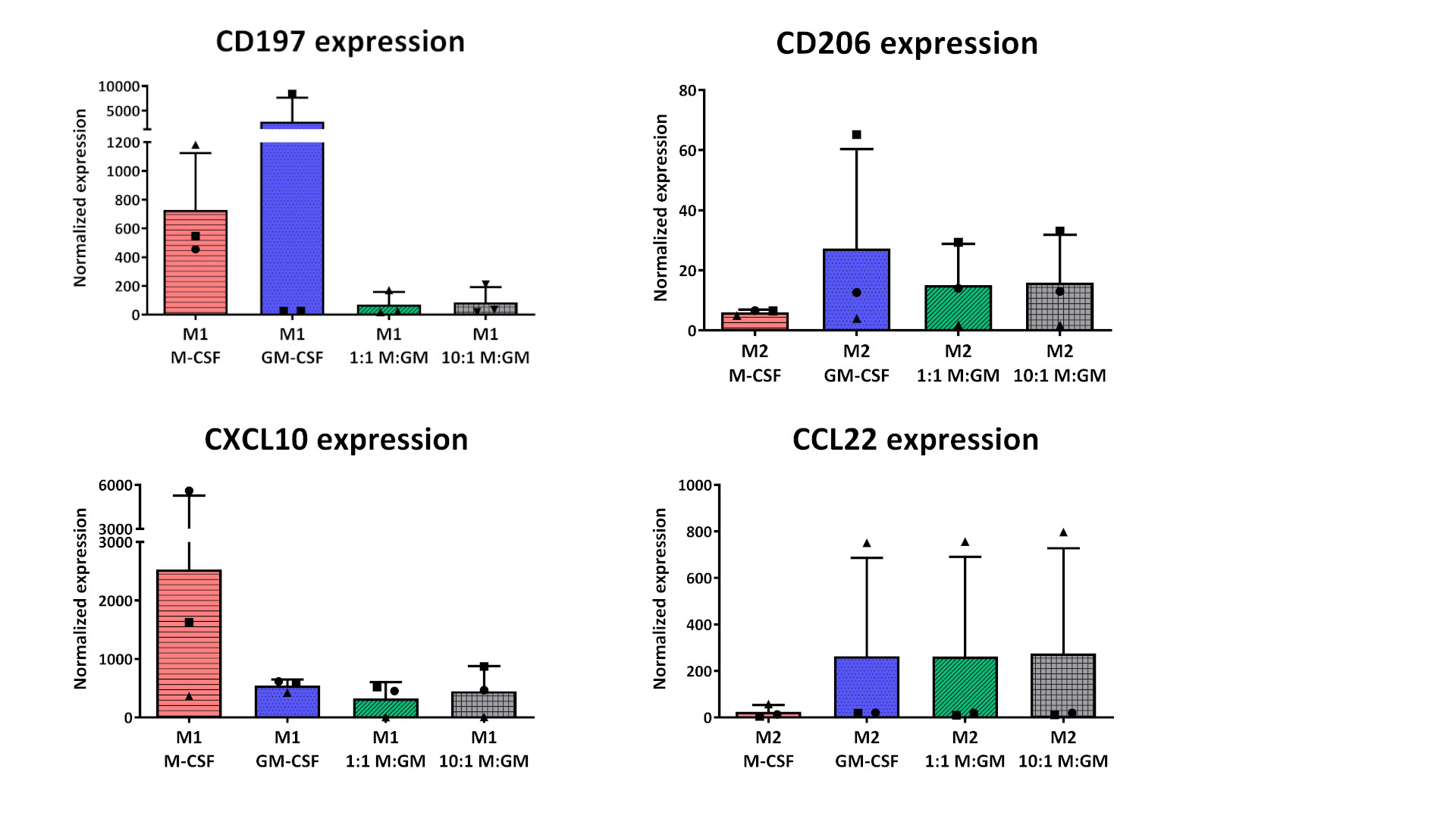


**Supplementary Figure S3**. Gene expression of primary macrophages obtained with different monocyte-to-macrophage differentiation conditions, upon stimulation to M1 or M2 phenotypes. Blood-derived monocytes were supplemented with either 20 ng/mL M-CSF (“M-CSF”), 20 ng/mL GM-CSF (“GM-CSF”) or a combination of the factors with a 1:1 or a 10:1 ratio of M-CSF:GM-CSF (“1:1 M:GM” and “10:1 M:GM”, respectively). Upon differentiation, macrophages were polarized for 24 hours into M1- or M2-like cells, then gene expression of M1 stimulated cells was analyzed with M1 markers CD197 and CXCL10, and M2 stimulated cells were analyzed for M2 markers CD206 and CCL22. GAPDH was used as reference gene. N=3, duplicate measurements. Error bars represent standard deviation (Kruskal-Wallis test).


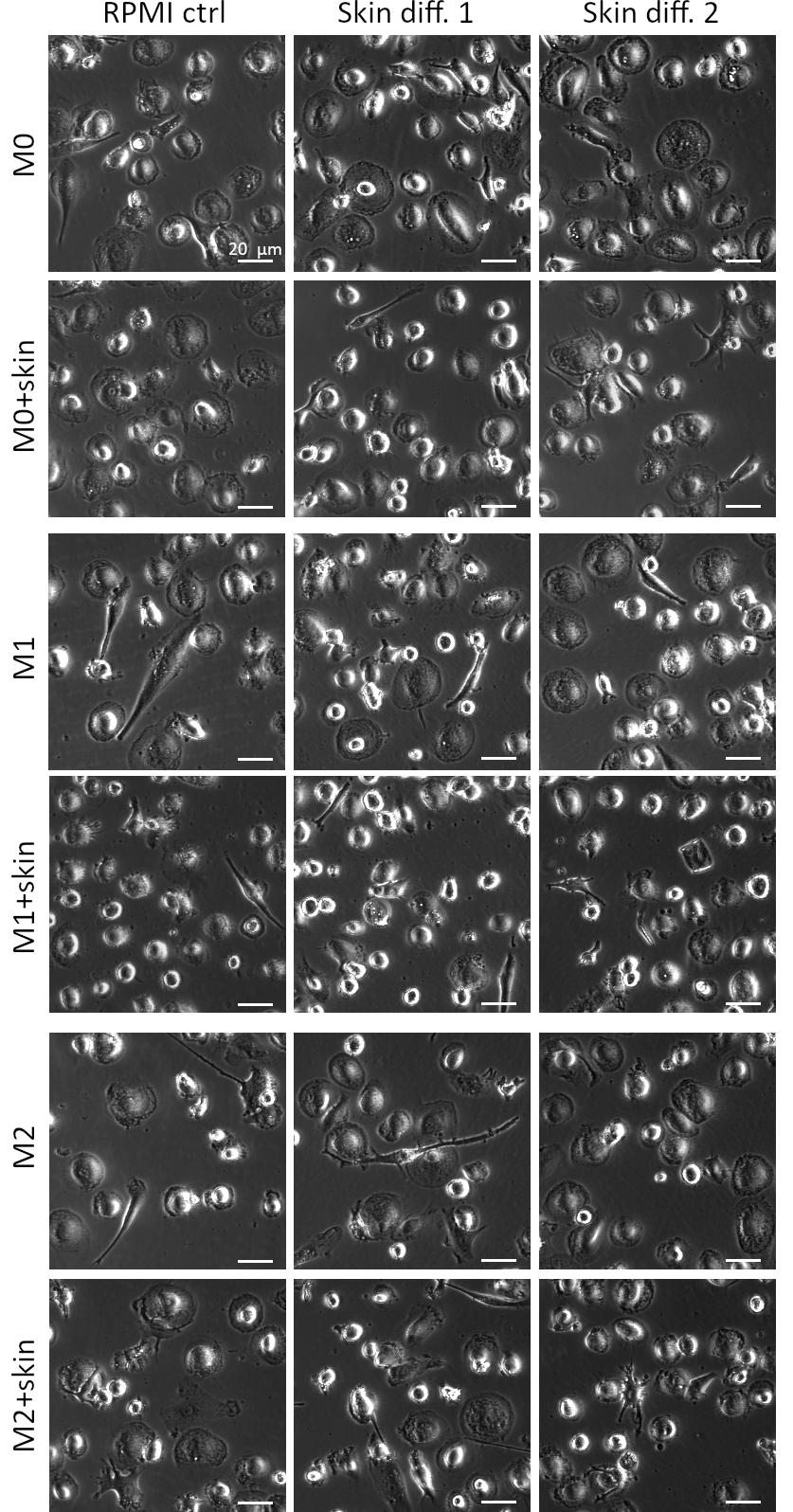


**Supplementary Figure S4**. Morphology of primary macrophages cultured in RPMI control (“RPMI ctrl”), skin differentiation 1 (“Skin diff. 1”) or skin differentiation medium 2 (“Skin diff. 2”), in presence ("M0+skin"; "M1+skin"; "M2+skin") or absence (“M0”; "M1"; "M2") of skin as a co-culture. Pictures were taken 24 hours after culture start. Scale bars: 20 µm.


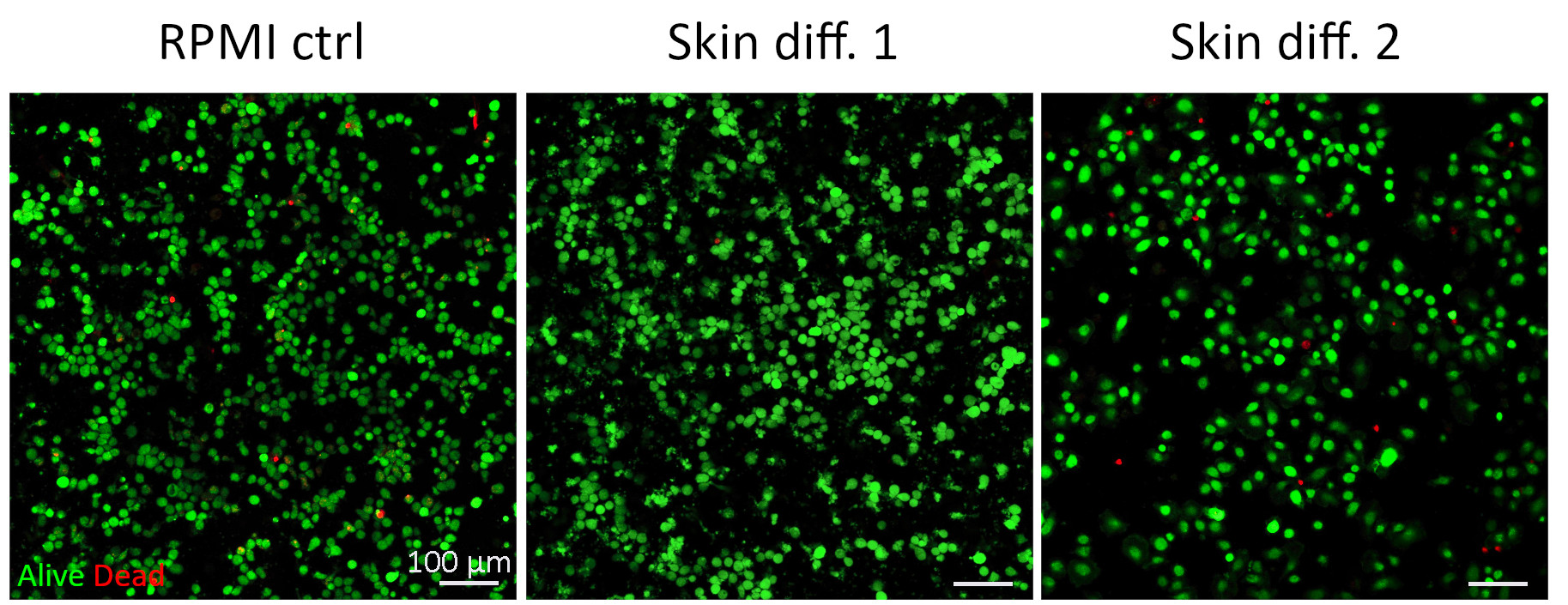


**Supplementary Figure S5.** Influence of culture medium on primary macrophages attachment to the surface of collagen gels. Representative images of primary macrophages seeded on top of collagen hydrogels and cultured for 48 hours in skin differentiation 1 (“Skin diff. 1”), skin differentiation 2 (“Skin diff. 2”) and RPMI control medium (“RPMI ctrl”). Images show a top view of each condition upon macrophages live/dead staining, 1 day after seeding the same number of cells per condition, corresponding to 75’000 cells each gel. Scale bars: 100 µm.

**Supplementary Table S1.** List of all components present in the media under investigation.

| [g/L] | **RPMI control medium** | **Skin differentiation 2 medium** | |
| --- | --- | --- | --- |
| **Inorganic salts** | | | |
| Ca(NO_3_)_2_•4H_2_O | 0.1 | | - |
| MgSO_4_ | 0.4884 | | 0.0732525 |
| KCl | 0.4 | | 0.356 |
| NaHCO_3_ | 2 | | 3.069 |
| NaCl | 6 | | 6.69975 |
| Na_2_HPO_4_ | 0.8 | | 0.11726 |
| CaCl_2_ | - | | 0.15 |
| CaCl_2_•2H_2_O | - | | 0.011025 |
| Fe(NO_3_)_3_• 9H_2_O | - | | 0.000075 |
| CuSO_4_• 5H_2_O | - | | 0.000000625 |
| FeSO_4_• 7H_2_O | - | | 0.0002085 |
| MgCl•6H_2_O | - | | 0.03075 |
| ZnSO_4_• 7H_2_O | - | | 0.00021575 |
| **Amino acids** | | | |
| L-Alanine | - | | 0.00225 |
| L-Arginine | 0.2 | | - |
| L-Arginine•HCl | - | | 0.11575 |
| L-Asparagine | 0.05 | | - |
| L-Asparagine•H_2_O | - | | 0.0037525 |
| L-Aspartic Acid | 0.02 | | 0.003325 |
| L-Cystine•2HCl | 0.0652 | | 0.04695 |
| L-Cystine•HCl•H_2_O | - | | 0.00875 |
| L-Glutamic Acid | 0.02 | | 0.003675 |
| Glycine | 0.01 | | 0.0243775 |
| L-Histidine | 0.015 | | - |
| L-Histidine•HCl•H_2_O | - | | 0.0315 |
| L-Histidine•3HCl•H_2_O | - | | 0.00524 |
| Hydroxy-L-Proline | 0.02 | | - |
| L-Isoleucine | 0.05 | | 0.0886 |
| L-Leucine | 0.05 | | 0.082025 |
| L-Lysine•HCl | 0.04 | | 0.118625 |
| L-Methionine | 0.015 | | 0.02362 |
| L-Phenylalanine | 0.015 | | 0.05074 |
| L-Proline | 0.02 | | 0.008625 |
| L-Serine | 0.03 | | 0.034125 |
| L-Threonine | 0.02 | | 0.074225 |
| L-Tryptophan | 0.005 | | 0.01251 |
| L-Tyrosine•2Na•2H_2_O | 0.02883 | | 0.0797875 |
| L-Valine | 0.02 | | 0.073425 |
| **Vitamins** | | | |
| D-Biotin | 0.0002 | | 0.000001825 |
| Choline Chloride | 0.003 | | 0.00649 |
| Folic Acid | 0.001 | | 0.00333 |
| myo-Inositol | 0.035 | | 0.0099 |
| Niacinamide | 0.001 | | 0.00300925 |
| p-Amino Benzoic Acid | 0.001 | | - |
| D-Pantothenic Acid•½Ca | 0.00025 | | 0.00312 |
| Pyridoxine•HCl | 0.001 | | 0.0030455 |
| Riboflavin | 0.0002 | | 0.0003095 |
| Thiamine•HCl | 0.001 | | 0.003085 |
| Vitamin B-12 | 0.000005 | | 0.00034 |
| **Other** | | | |
| D-Glucose | 2 | | 1.2005 |
| Glutathione (reduced) | 0.001 | | - |
| Phenol Red•Na | 0.0053 | | 0.01225 |
| Pyruvic acid•Na | - | | 0.11 |
| Hypoxanthine | - | | 0.00102 |
| Linoleic Acid | - | | 0.000021 |
| Putrescine•HCl | - | | 0.00004025 |
| Thioctic Acid | - | | 0.0000525 |
| Thymidine | - | | 0.0001825 |
| **Supplements** | | | |
| PSN antibiotic mixture | 1 % | | 1 % |
| L-glut | 1 % | | 1 % |
| Fetal bovine serum | 10 % | | 1.25 % |
| Hydrocortisone | - | | 1 µM |
| Isoproterenol | - | | 1 µM |
| Insulin | - | | 0.1 µM |
| KGF | - | | 2 ng/mL |
| L-carnitine | - | | 0.1 µM |
| L-serine | - | | 0.01 M |
| Ascorbic acid-2-phosphate | - | | 50 µg/mL |
